# Increased stressful impact among general population in mainland China amid the COVID-19 pandemic: A nationwide cross-sectional study conducted after Wuhan city’s travel ban was lifted

**DOI:** 10.1177/0020764020935489

**Published:** 2020-06-22

**Authors:** Zheng Feei Ma, Yutong Zhang, Xiaoqin Luo, Xinli Li, Yeshan Li, Shuchang Liu, Yingfei Zhang

**Affiliations:** 1 Department of Health and Environmental Sciences, Xi’an Jiaotong-Liverpool University, Suzhou, China; 2 Jinzhou Medical University, Jinzhou, China; 3 Department of Nutrition and Food Safety, School of Public Health, Xi’an Jiaotong University, Xi’an, China; 4 Department of Nutrition and Food Hygiene, School of Public Health, Medical College of Soochow University, Suzhou, China; 5 Department of Respiratory and Critical Care Medicine, The Second People’s Hospital of Wuhu, Wuhu, China; 6 School of Biosciences, University of Nottingham, Loughborough, UK; 7 Mathematics Teaching and Research Office, Public Basic College, Jinzhou Medical University, Jinzhou, China

**Keywords:** Coronavirus, COVID-19, mental health, lifestyle changes, China

## Abstract

**Objectives:**

Our study aimed to determine the impact of the COVID-19 pandemic on psychological responses and lifestyle changes among the general population in mainland China following the re-opening of the Wuhan city.

**Methods:**

A cross-sectional survey was conducted in April 2020. Participants of Chinese nationality aged ⩾ 18 years were asked to complete a modified validated Chinese version of a questionnaire regarding the impact of event scale (IES), family and social support, mental health–related lifestyle changes, and indicators of negative mental health impacts.

**Results:**

A total of 728 participants (i.e., 217 males and 511 females) completed the questionnaire. The mean age of the participants was 32.9 ± 10.4 years, with a majority of them (92.2%) having a higher educational qualification level. The overall mean IES in participants was 21.5 ± 7.0, reflecting mild stressful impact (i.e., following the re-opening of the Wuhan city); 25.5% of the participants had an IES score ⩾ 26. Being females and married were significantly associated with a higher mean IES score. The overall mean scores for intrusion and avoidance score scales in participants were 9.4 ± 3.7 and 12.1 ± 4.2, respectively.

**Conclusions:**

The COVID-19 pandemic was associated with increased stressful impact in our participants following the re-opening of the Wuhan city when compared with our previous study, which should not be taken lightly.

## Introduction

Since January 2020, the COVID-19 epidemic, which is an infectious disease caused by the SARS-CoV-2 infection, has been declared as a public health emergency of international concern (Zheng, 2020). SARS-CoV-2 has been identified as one of the members of the coronavirus family, which can cause infections in both humans and animals (Yang et al., 2020). Human–human transmission has been reported through the SARS-CoV-2 virus-laden respiratory droplets (Huang et al., 2020). In addition, the transmissibility of the COVID-19 has been estimated to be 4.1 based on its reproductive numbers, indicating that on average, every confirmed case of COVID-19 will create up to a maximum of 4 new confirmed cases of COVID-19 (Wang, Pan, Wan, Tan, Xu, Ho, & Ho, 2020).

In order to control the COVID-19 pandemic in China, the Chinese government had implemented several restrictive measures including the lockdown of the Wuhan city (Chen et al., 2020). Currently, as of 7 May 2020, there are 84,409 confirmed cases of COVID-19, which has led to 4,643 deaths in China (https://www.who.int/emergencies/diseases/novel-coronavirus-2019/situation-reports/). Although such measures imposed by the Chinese government were effective in containing the COVID-19 spread, these measures have disrupted the jobs and lives of the general population, which might have had some impacts on their well-being and health (Lima et al., 2020). This is because such measures have placed the general population in isolation and subsequently, this might trigger a wide range of psychological issues including depression, stress and anxiety, especially in vulnerable groups.

Previously, we had reported some immediate impacts of the COVID-19 pandemic on the quality of life and mental health outcomes among the Chinese local residents living in the Northeast China 1 week after the Wuhan city was locked down on 23 January 2020 for 11 weeks (Zhang & Ma, 2020). However, it is unclear if the impact of COVID-19 pandemic on the psychological responses among the general population was similar across different provinces in mainland China. This is especially after the travel restrictions in the Wuhan city were lifted on 8 April 2020. Although there are some studies that reported the psychological impact of the COVID-19 pandemic among the Chinese populations, most of them, including our previous study, were conducted before the travel restrictions in the Wuhan city were lifted (Qiu et al., 2020; Wang, Pan, Wan, Tan, Xu, McIntyre, et al., 2020; Zhang & Ma, 2020).

Therefore, the aim of the study was to investigate the psychological impact of the COVID-19 pandemic among the general population in mainland China immediately after the Wuhan city was re-opened on 8 April 2020 by the Chinese government. The results of our study will be used to deepen our understanding of the impact of COVID-19 pandemic on psychological responses and lifestyle changes among general population in mainland China. In addition, appropriate health education programmes can be formulated based on the findings of our previous and current study, which were conducted during different stages of the COVID-19 pandemic (Zhang & Ma, 2020).

## Methods

A cross-sectional survey was conducted between 9 April 2020 and 30 April 2020. To be eligible for the study, they must be non-pregnant Chinese nationality aged ⩾ 18 years who have lived in mainland China amid the COVID-19 pandemic. In addition, they must be able to give verbal informed consent prior to the study enrolment. Participants were recruited by snowball and convenience sampling methods. Recruitment strategies used included the use of social media and word of mouth. No monetary incentives were given for participating in the study. To ensure data integrity, the participants’ confidentiality was maintained throughout the data collection and analysis. Participants were asked to voluntarily participate in the research study. Our research study was approved by the Ethics Committee of the Jinzhou Medical University (ref. no. JYDLL2020002). In addition, our study research protocols were conducted in accordance with The Code of Ethics of the World Medical Association (Declaration of Helsinki).

### Impact of event scale

An online questionnaire of modified and validated Chinese version of impact of event scale (IES) including questions on socio-demographic variables (e.g., age, sex and current residence) was distributed to participants via WeChat and phone interviews. The IES questionnaire consisted of 15 questions with a Cronbach’s alpha value of 0.8 (Lau et al., 2006; Zhang & Ma, 2020). It was used to assess the excessive panic and anxiety levels of the participants amid the pandemic (i.e., following the re-opening of the Wuhan city). There were two subscales (i.e., avoidance and intrusive) in the IES questionnaire. Response options for each question included the following: not at all = 0, rarely = 1, sometimes = 3 and often = 5. Participants with an IES score of ⩾ 26 was considered to experience moderate-to-severe stressful impact.

### Impact of COVID-19 on family and social support

In addition, participants were asked to complete a modified and validated 5-item questionnaire on the impact of the COVID-19 pandemic on family and social support they received (Cronbach’s alpha value of 0.9) following the re-opening of the Wuhan city. These questions included the following: support from family members, sharing feelings with other family members, support from friends, caring for family members’ feelings and sharing feelings with others (Lau et al., 2006; Zhang & Ma, 2020). There were five response options for these questions: much decreased = 1, decreased = 2, same as before = 3, increased = 4 and much increased = 5. A higher score was used to suggest a higher social and family support received by participants amid the COVID-19 (Lau et al., 2006; Zhang & Ma, 2020).

### Impact on mental health–related lifestyle changes

The online questionnaire also consisted of four questions which were about the mental health–related lifestyle changes (Cronbach’s alpha value of 0.8). Participants were asked to indicate if they experienced any mental health–related lifestyle changes amid the COVID-19 pandemic (i.e., following the re-opening of the Wuhan city) including the following: paying attention to mental health, time to rest, relax and exercise by selecting one of the following response options: much decreased = 1, decreased = 2, same as before = 3, increased = 4 or much increased = 5). Their scores were computed using the Mental Health Lifestyle Scale (MHLSS). A higher MHLSS score was used to suggest more favourable changes in their mental health–related lifestyle (Lau et al., 2006; Zhang & Ma, 2020).

### Other indicators of negative mental health impacts

Six modified and validated questions related to the negative mental health impacts experienced by the participants amid the COVID-19 pandemic (i.e., following the re-opening of the Wuhan city) were also included in the last part of the online questionnaire with a Cronbach’s alpha value of 0.9. These questions included the following: increased stress from work, financial stress, stress from home, horrified feelings, and feeling apprehensive or helpless due to the COVID-19 pandemic (i.e., following the re-opening of the Wuhan city). Participants were asked to rate their response (much decreased = 1, decreased = 2, same as before = 3, increased = 4 or much increased = 5). A higher score was used to indicate more severe negative mental health impact (Lau et al., 2006; Zhang & Ma, 2020).

### Statistical analysis

Statistical analysis was conducted using SPSS version 16.0 (SPSS, Chicago, IL). The results of continuous and categorical variables were reported as mean ± standard deviation (*SD*) and frequency (percentage, %), respectively. A Chi-square test was used to determine if there was a significant relationship between two categorical variables. An unpaired t-test was employed to assess if there was a difference in variables’ scores between different categorical variables including sex, education level and religion. General linear model (GLM) multivariate analysis was performed to examine the difference in dependent variables among independent variables including regions. In addition, multiple linear regression analysis was used to examine the effect of socio-demographic factors on IES scores. A *p*-value < 0.05 was used to indicate statistical significance.

## Results

### Participant characteristics

Of the 1000 participants who were invited, 728 participants (i.e., 217 males and 511 females) were recruited into the study with a response rate of 72.8% (Table 1). Those who rejected the study invitation (*n* = 272) provided the following reasons: not interested (66.2%) and no time to complete the questionnaire (33.8%). The mean age of the participants was 32.9 ± 10.4 years, with a majority of them (41.3%) aged between 18 and 29 years. The mean BMI of the participants was 22.5 ± 3.2 kg/m^2^, which was categorised as normal BMI range. A majority of the participants (92.2%) had a higher level of education. There were 59.3% of the participants who indicated that they were married at the time the study was conducted. A majority of the participants were from East China (40.9%), followed by Northwest China (23.8%), North China (13.7%), South Central China (11.1%), Northeast China (6.0%) and Southwest China (4.4%). In terms of employment status, 58.5% of the participants had a full-time job, followed by student status (30.8%) and part-time job (10.7%); 94.1% of the participants were of Han ethnicity, and a majority of the participants (91.5%) reported no religious belief. None of the participants reported that they were diagnosed with COVID-19 or that their family members/friends were tested positive for the COVID-19 when our study was carried out.

**Table 1.** Socio-demographic characteristics of the participants.

	All (*n* = 728)	Females (*n* = 511)	Males (*n* = 217)	P-value
Age (years)	32.9 ± 10.4	32.6 ± 10.2	33.7 ± 10.9	0.196
BMI (kg/m²)	22.5 ± 3.2	21.8 ± 3.0	24.0 ± 3.3	**<** **0.001**
Education level, *n* (%)				
*Secondary school*	57 (7.8)	33 (6.5)	24 (11.1)	**0.034**
*Higher educational qualification*	671 (92.2)	478 (93.5)	193 (88.9)	
Marital status, *n* (%)				
*Single/Divorced*	296 (40.7)	203 (39.7)	93 (42.9)	0.431
*Married*	432 (59.3)	308 (60.3)	124 (57.1)	
Region, *n* (%)				
*East*	298 (40.9)	226 (44.2)	72 (33.2)	**<** **0.001**
*North*	100 (13.7)	57 (11.2)	43 (19.8)	
*Northeast*	44 (6.0)	32 (6.3)	12 (5.5)	
*Northwest*	173 (23.8)	129 (25.2)	44 (20.3)	
*South Central*	81 (11.1)	42 (8.2)	39 (18.0)	
*Southwest*	32 (4.4)	25 (4.9)	7 (3.2)	
Employment status, *n* (%)				
*Full-time*	426 (58.5)	305 (59.7)	121 (55.8)	**0.038**
*Part-time*	78 (10.7)	45 (8.8)	33 (15.2)	
*Students*	224 (30.8)	161 (31.5)	63 (29)	
Ethnicity, *n* (%)				
*Han*	685 (94.1)	482 (94.3)	203 (93.5)	0.684
*Others*	43 (5.9)	29 (5.7)	14 (6.5)	
Religion, *n* (%)				
*No*	666 (91.5)	466 (91.2)	200 (92.2)	0.667
*Yes*	62 (8.5)	45 (8.8)	17 (7.8)	

BMI: body mass index.

### IES

The overall mean IES score in participants was 21.5 ± 7.0, reflecting mild stressful impact due to the COVID-19 pandemic (i.e., following the re-opening of the Wuhan city). Being female and married were associated with a higher mean IES score (all *p* < .05) (Tables 1 and 2). Other socio-demographic variables including region, age group, education level, religion and ethnicity were not associated with the IES score (all *p* > .05). Overall, 25.5% of the participants had an IES score ⩾ 26. There were significantly higher percentages of the participants who were married and working full-time with an IES score ⩾ 26 (all *p* < .05). Other socio-demographic variables including sex, age group, region, education level, religion and ethnicity were not associated with the percentage of the participants with IES ⩾ 26. The overall mean scores for intrusion and avoidance score scales in participants were 9.4 ± 3.7 and 12.1 ± 4.2, respectively. Only the mean intrusive score in participants who were female and married was significantly higher than that of the participants who were male and single (9.6 and 9.6 vs. 8.8 and 8.9, respectively) (*p* = .004 and 0.011, respectively). There was no association between the mean intrusive score and other demographic factors including age group, region, education level, religion, employment status and ethnicity (*p* > .05). Participants who were married had a significantly higher avoidance scale score than participants who were single (12.4 vs. 11.7) (*p* = .028). There was no association between the mean avoidance score and other demographic factors including sex, age group, region, education level, religion, employment status and ethnicity (all *p* > .05).

**Table 2.** Psychological responses and lifestyle changes by gender and region.

	Sex (*n* = 728)		*P*-value	Region (*n* = 728)						*P*-value
	Females (*n* = 511)	Males (*n* = 217)		North (*n* = 100)	Northeast (*n* = 44)	East (*n* = 298)	South Central (*n* = 81)	Southwest (*n* = 32)	Northwest (*n* = 173)	
IES	21.9 ± 6.9	20.4 ± 7.0	**0.008**	21.4 ± 9.3	23.4 ± 6.4	21.2 ± 6.7	21.2 ± 7.1	21.7 ± 6.5	21.5 ± 6.0	0.560
*IES* *⩾* *26, n (%)*	141 (27.6)	45 (20.7)	0.052	29 (29.0)	19 (43.2)	69 (23.2)	19 (23.5)	8 (25.0)	42 (24.3)	0.107
*Intrusive scale score*	9.6 ± 3.6	8.8 ± 3.7	**0.004**	9.6 ± 4.6	10.6 ± 3.6	9.1 ± 3.5	9.3 ± 3.6	9.6 ± 3.6	9.4 ± 3.4	0.188
*Avoidance scale score*	12.3 ± 4.2	11.7 ± 4.2	0.057	11.8 ± 5.4	12.8 ± 3.7	12.2 ± 4.1	11.8 ± 4.5	12.1 ± 4.0	12.2 ± 3.7	0.832
**Family and social support**										
*Getting support from friends*	3.5 ± 0.8	3.4 ± 0.8	0.059	3.4 ± 0.9	3.4 ± 0.8	3.5 ± 0.8	3.5 ± 0.8	3.5 ± 0.6	3.5 ± 0.7	0.742
*Getting support from family members*	3.7 ± 0.9	3.6 ± 0.9	0.165	3.6 ± 1.0	3.8 ± 0.8	3.6 ± 0.9	3.7 ± 0.9	3.8 ± 0.9	3.8 ± 0.9	0.392
*Shared feelings with family members*	3.6 ± 0.8	3.5 ± 0.9	**0.010**	3.5 ± 1.0	3.8 ± 0.7	3.6 ± 0.8	3.6 ± 0.9	3.8 ± 0.8	3.5 ± 0.8	0.236
*Shared feelings with others when feeling blue*	3.6 ± 0.9	3.4 ± 0.9	**0.004**	3.5 ± 0.9	3.5 ± 1.0	3.5 ± 0.8	3.5 ± 0.9	3.8 ± 0.8	3.4 ± 0.9	0.281
*Caring for family members’ feelings*	3.3 ± 0.9	3.3 ± 0.9	0.280	3.4 ± 1.0	3.4 ± 1.0	3.4 ± 0.8	3.3 ± 1.0	3.6 ± 0.7	3.2 ± 0.9	0.095
**Awareness and lifestyles**										
*Pay attention to mental health*	3.7 ± 0.7	3.6 ± 0.8	0.199	3.6 ± 0.8	3.9 ± 0.8	3.6 ± 0.8	3.7 ± 0.8	3.6 ± 0.8	3.6 ± 0.7	0.284
*Time spent to rest*	3.8 ± 0.7	3.8 ± 0.8	0.913	3.7 ± 0.8	3.9 ± 0.7	3.8 ± 0.7	3.9 ± 0.7	3.7 ± 0.7	3.9 ± 0.6	0.376
*Time spent to relax*,	3.7 ± 0.7	3.7 ± 0.8	0.614	3.7 ± 0.9	3.8 ± 0.8	3.7 ± 0.7	3.6 ± 0.7	3.6 ± 0.8	3.7 ± 0.7	0.942
*Time spent to exercise*	3.5 ± 0.7	3.4 ± 0.6	0.115	3.5 ± 1.0	3.6 ± 0.7	3.5 ± 0.7	3.5 ± 0.7	3.5 ± 0.8	3.5 ± 0.7	0.879
**Negative health impacts**										
*Increased stress from work*	1.6 ± 0.7	1.7 ± 0.8	**0.005**	2.0 ± 0.9	1.4 ± 0.8	1.5 ± 0.7	1.8 ± 0.8	1.9 ± 0.8	1.5 ± 0.6	**<** **0.001**
*Increased financial stress*	3.2 ± 0.9	3.0 ± 1.0	**0.011**	3.1 ± 1.0	3.6 ± 1.0	3.2 ± 0.9	3.1 ± 0.9	3.3 ± 1.1	3.1 ± 0.9	**0.021**
*Increased stress from home*	3.2 ± 0.9	3.1 ± 1.0	**0.028**	3.0 ± 0.9	3.4 ± 0.8	3.2 ± 0.9	3.0 ± 0.9	3.5 ± 0.9	3.2 ± 0.9	0.067
*Feel horrified due to the COVID-19*	3.6 ± 0.8	3.4 ± 1.0	**0.032**	3.2 ± 1.0	3.5 ± 0.7	3.5 ± 1.0	3.5 ± 1.0	3.5 ± 0.9	3.6 ± 0.9	**0.030**
*Feel apprehensive due to the COVID-19*	3.4 ± 0.7	3.5 ± 0.9	**0.424**	3.4 ± 0.9	3.3 ± 0.9	3.4 ± 0.8	3.6 ± 0.7	3.4 ± 0.8	3.5 ± 0.7	0.133
*Feel helpless due to the COVID-19*	3.3 ± 0.7	3.4 ± 0.8	0.356	3.3 ± 0.8	3.4 ± 0.6	3.2 ± 0.7	3.4 ± 0.8	3.2 ± 0.8	3.3 ± 0.7	0.257

IES: impact of event scale.

### Impact on family and social support

After the travel restrictions in the Wuhan city were lifted, female participants reported that they were significantly more likely to share their feelings with their family members and others when feeling blue (3.6 and 3.6, respectively) than males (3.5 and 3.4, respectively) (*p* = .010 and 0.004, respectively) (Table 2). Participants aged 40–49 years were significantly more likely to get increased support from friends than other age groups (*p* = .001) (Table 3). On the other hand, participants aged 18–29 years were significantly more likely to share their feelings with others when feeling blue than other age groups (*p* = .015). Participants with a higher educational qualification level were significantly more likely to receive increased support from friends and family members than participants with a lower educational qualification level (*p* = .035 and 0.023, respectively).

**Table 3.** Psychological responses and lifestyle changes by age groups, education levels and religion beliefs.

	Age group (years) (*n* = 728)				*P*-value	Education level (*n* = 728)		*P*-value	Religion (*n* = 728)		*P*-value
	18–29 (*n* = 301)	30–39 (*n* = 276)	40–49 (*n* = 78)	⩾ 50 (*n* = 73)		Secondary school (*n* = 57)	Higher educational qualification (*n* = 671)		No (*n* = 666)	Yes (*n* = 62)	
IES	20.8 ± 6.4	21.9 ± 7.3	21.2 ± 7.7	22.8 ± 7.5	0.109	22.8 ± 8.0	21.4 ± 6.9	0.144	21.3 ± 6.9	22.8 ± 8.4	0.125
*IES* *⩾* *26, n (%)*	65 (21.6)	77 (27.9)	19 (24.4)	25 (34.2)	0.101	20 (35.1)	166 (24.7)	0.085	165 (24.8)	21 (33.9)	0.116
*Intrusive scale score*	9.1 ± 3.5	9.5 ± 3.7	9.3 ± 4.2	10.1 ± 3.8	0.151	10.1 ± 3.9	9.3 ± 3.6	0.107	9.3 ± 3.6	10.0 ± 4.1	0.157
*Avoidance scale score*	11.8 ± 4.0	12.5 ± 4.3	11.9 ± 4.2	12.6 ± 4.6	0.163	12.7 ± 5.1	12.1 ± 4.1	0.393	12.1 ± 4.2	12.8 ± 4.7	0.188
**Family and social support**											
*Getting support from friends*	3.4 ± 0.8	3.5 ± 0.8	3.7 ± 0.9	3.2 ± 0.9	**0.001**	3.3 ± 0.9	3.5 ± 0.8	**0.035**	3.5 ± 0.8	3.5 ± 0.8	0.670
*Getting support from family members*	3.6 ± 0.9	3.7 ± 0.9	3.8 ± 1.0	3.6 ± 1.0	0.275	3.4 ± 1.2	3.7 ± 0.9	**0.023**	3.7 ± 0.9	3.6 ± 1.1	0.365
*Shared feelings with family members*	3.5 ± 0.8	3.6 ± 0.8	3.7 ± 0.9	3.7 ± 0.8	0.325	3.5 ± 1.1	3.6 ± 0.8	0.282	3.6 ± 0.8	3.5 ± 1.0	0.179
*Shared feelings with others when feeling blue*	3.6 ± 0.8	3.5 ± 0.9	3.5 ± 1.0	3.2 ± 0.9	**0.015**	3.3 ± 1.1	3.5 ± 0.8	0.066	3.5 ± 0.9	3.5 ± 0.8	0.645
*Caring for family members’ feelings*	3.3 ± 0.8	3.3 ± 0.9	3.4 ± 1.1	3.1 ± 1.0	0.237	3.1 ± 1.0	3.3 ± 0.9	0.123	3.3 ± 0.9	3.2 ± 0.9	0.117
**Awareness and lifestyles**											
*Pay attention to mental health*	3.6 ± 0.8	3.6 ± 0.7	3.6 ± 0.8	3.7 ± 0.8	0.915	3.6 ± 0.9	3.6 ± 0.8	0.854	3.6 ± 0.8	3.7 ± 0.8	0.623
*Time spent to rest*	3.8 ± 0.7	3.8 ± 0.7	3.9 ± 0.7	3.9 ± 0.7	0.406	3.8 ± 0.9	3.8 ± 0.7	0.466	3.8 ± 0.7	3.8 ± 0.8	0.729
*Time spent to relax*,	3.7 ± 0.8	3.7 ± 0.7	3.7 ± 0.7	3.7 ± 0.7	0.721	3.5 ± 0.8	3.7 ± 0.7	**0.026**	3.7 ± 0.7	3.6 ± 0.8	0.449
*Time spent to exercise*	3.4 ± 0.7	3.5 ± 0.7	3.5 ± 0.8	3.6 ± 0.7	0.563	3.4 ± 0.9	3.5 ± 0.7	0.556	3.5 ± 0.7	3.6 ± 0.9	0.330
**Negative health impacts**											
*Increased stress from work*	1.7 ± 0.8	1.6 ± 0.7	1.5 ± 0.8	1.5 ± 0.7	**0.007**	1.8 ± 0.8	1.6 ± 0.7	**0.034**	1.6 ± 0.7	1.8 ± 0.8	0.137
*Increased financial stress*	3.1 ± 1.0	3.3 ± 0.9	3.2 ± 0.9	3.4 ± 0.9	**0.028**	3.2 ± 1.1	3.2 ± 0.9	0.710	3.2 ± 0.9	3.3 ± 1.0	0.171
*Increased stress from home*	3.1 ± 0.9	3.2 ± 0.9	3.3 ± 0.9	3.4 ± 0.8	**0.015**	3.1 ± 1.0	3.2 ± 0.9	0.291	3.2 ± 0.9	3.2 ± 0.8	0.628
*Feel horrified due to the COVID-19*	3.4 ± 0.9	3.6 ± 0.8	3.4 ± 1.0	3.6 ± 0.8	0.059	3.2 ± 1.1	3.5 ± 0.9	**0.010**	3.5 ± 0.9	3.4 ± 0.8	0.178
*Feel apprehensive due to the COVID-19*	3.4 ± 0.8	3.5 ± 0.8	3.4 ± 0.8	3.4 ± 0.8	0.247	3.5 ± 1.0	3.4 ± 0.8	0.350	3.4 ± 0.8	3.5 ± 0.8	0.220
*Feel helpless due to the COVID-19*	3.2 ± 0.7	3.3 ± 0.7	3.2 ± 0.8	3.5 ± 0.7	**0.025**	3.6 ± 0.8	3.3 ± 0.7	**0.001**	3.3 ± 0.7	3.3 ± 0.8	0.724

IES: impact of event scale.

In addition, participants who were married reported a significantly increased support from friends and family members than participants who were single (*p* = .006 and 0.002, respectively) (Table 4). Also, participants who were married were significantly more likely to share their feelings with family members than participants who were single (3.7 vs. 3.5) (*p* = .004) (Table 4). Participants who were students reported a significantly higher family and social support in terms of ‘shared feelings with other when feeling blue’ and ‘caring for family members’ feelings’ than participants with full-time and part-time jobs (*p* = .008 and 0.035).

**Table 4.** Psychological responses and lifestyle changes by marital status, job and ethnicity.

	Marital status (*n* = 728)		*P*-value	Job (*n* = 728)			*P*-value	Ethnicity (*n* = 728)		*P*-value
	Married (*n* = 432)	Single (*n* = 269)		Full-time (*n* = 426)	Part-time (*n* = 78)	Students (*n* = 224)		Han (*n* = 685)	Others (*n* = 43)	
IES	22 ± 7.2	20.6 ± 6.6	**0.008**	21.7 ± 7.4	22.5 ± 7.2	20.7 ± 6.0	0.107	21.4 ± 7.0	22.4 ± 7.6	0.348
*IES* *⩾* *26, n (%)*	125 (28.9)	61 (20.6)	**0.011**	116 (27.2)	29 (37.2)	41 (18.3)	**0.002**	173 (25.3)	13 (30.2)	0.468
*Intrusive scale score*	9.6 ± 3.8	8.9 ± 3.5	**0.011**	9.4 ± 3.8	9.9 ± 3.7	9.0 ± 3.3	0.168	9.3 ± 3.7	9.6 ± 4.0	0.643
*Avoidance scale score*	12.4 ± 4.3	11.7 ± 4.1	**0.028**	12.2 ± 4.4	12.6 ± 4.4	11.7 ± 3.8	0.174	12.1 ± 4.2	12.8 ± 4.3	0.248
**Family and social support**										
*Getting support from friends*	3.5 ± 0.8	3.4 ± 0.8	**0.006**	3.5 ± 0.9	3.3 ± 0.7	3.4 ± 0.7	0.136	3.5 ± 0.8	3.4 ± 1.0	0.462
*Getting support from family members*	3.8 ± 0.9	3.6 ± 0.9	**0.002**	3.7 ± 0.9	3.7 ± 1.1	3.7 ± 0.7	0.788	3.7 ± 0.9	3.3 ± 1.1	**0.002**
*Shared feelings with family members*	3.7 ± 0.8	3.5 ± 0.8	**0.004**	3.6 ± 0.9	3.5 ± 1.0	3.7 ± 0.7	0.073	3.6 ± 0.8	3.4 ± 1.0	0.170
*Shared feelings with others when feeling blue*	3.5 ± 0.9	3.6 ± 0.8	0.137	3.5 ± 0.9	3.3 ± 0.9	3.6 ± 0.8	**0.008**	3.5 ± 0.9	3.6 ± 1.0	0.524
*Caring for family members’ feelings*	3.3 ± 0.9	3.3 ± 0.8	0.776	3.3 ± 0.9	3.1 ± 0.9	3.4 ± 0.7	**0.035**	3.3 ± 0.9	3.3 ± 1.1	0.800
**Awareness and lifestyles**										
*Pay attention to mental health*	3.7 ± 0.7	3.6 ± 0.8	0.060	3.6 ± 0.8	3.6 ± 0.9	3.6 ± 0.7	0.991	3.6 ± 0.7	3.6 ± 1.0	0.514
*Time spent to rest*	3.9 ± 0.7	3.8 ± 0.7	0.058	3.8 ± 0.7	3.8 ± 0.9	3.8 ± 0.6	0.718	3.8 ± 0.7	3.6 ± 1.0	**0.022**
*Time spent to relax*,	3.7 ± 0.7	3.7 ± 0.8	0.453	3.7 ± 0.8	3.6 ± 0.8	3.8 ± 0.6	0.141	3.7 ± 0.7	3.7 ± 0.9	0.704
*Time spent to exercise*	3.5 ± 0.7	3.4 ± 0.8	**0.019**	3.5 ± 0.8	3.4 ± 0.8	3.6 ± 0.6	0.212	3.5 ± 0.7	3.4 ± 1.0	0.341
**Negative health impacts**										
*Increased stress from work*	1.5 ± 0.7	1.8 ± 0.8	**<** **0.001**	1.6 ± 0.7	1.7 ± 0.7	1.7 ± 0.8	0.418	1.6 ± 0.8	1.7 ± 0.7	0.639
*Increased financial stress*	3.3 ± 0.9	3.1 ± 1.0	**0.005**	3.2 ± 0.9	3.3 ± 0.9	3.1 ± 0.9	**0.021**	3.2 ± 0.9	3.4 ± 1.1	0.124
*Increased stress from home*	3.2 ± 0.9	3.1 ± 0.9	0.050	3.2 ± 0.9	3.3 ± 0.8	3.1 ± 0.9	**0.044**	3.2 ± 0.9	3.3 ± 0.9	0.391
*Feel horrified due to the COVID-19*	3.6 ± 0.9	3.4 ± 0.9	**0.008**	3.6 ± 0.9	3.5 ± 1.0	3.4 ± 0.8	**0.014**	3.5 ± 0.9	3.4 ± 0.9	0.250
*Feel apprehensive due to the COVID-19*	3.5 ± 0.8	3.3 ± 0.8	**0.015**	3.4 ± 0.8	3.7 ± 0.9	3.3 ± 0.7	**0.001**	3.4 ± 0.8	3.4 ± 0.9	0.559
*Feel helpless due to the COVID-19*	3.4 ± 0.7	3.2 ± 0.7	**0.005**	3.3 ± 0.7	3.5 ± 0.7	3.3 ± 0.7	0.068	3.3 ± 0.7	3.3 ± 0.9	0.960

IES: impact of event scale.

There were no significant differences in the questions regarding the impact of COVID-19 pandemic on family and family support for other socio-demographic variables including region and religion (all *p* > .05).

### Impact on mental health–related lifestyle changes

Following the re-opening of the Wuhan city, higher educational level was associated with increased spending of time to relax (*p* = .026) (Table 3). In addition, participants who were married were significantly more likely to spend more time exercising than participants who were single (3.5 vs. 3.4) (*p* = .019) (Table 4). In addition, participants who were of Han ethnicity were significantly more likely to spend more time to relax than participants of other ethnicities (3.8 vs. 3.6) (*p* = .022).

There were no significant differences in the questions regarding the impact of COVID-19 pandemic on mental health–related lifestyle changes for other socio-demographic variables including sex, region, age group, employment status and religion (all *p* > .05).

### Other indicators of negative mental health impacts

After the Wuhan city was re-opened, male participants reported that there was a significantly increased stress from work compared to female participants (1.7 vs. 1.6) (*p* = .005) (Table 2). On the other hand, female participants reported a significantly increased financial stress and stress from home (3.2 and 3.2, respectively) compared to male participants (3.0 and 3.1, respectively) (*p* = .011 and 0.028, respectively). In addition, female participants were also significantly more likely to feel horrified due to the COVID-19 pandemic (i.e., following the re-opening of Wuhan city) than male participants (3.6 vs. 3.4) (*p* = .032).

Although participants from Northeast China reported a significantly decreased stress from work compared to participants from other regions of China (*p* < .001), participants from Northeast China reported a significantly increased financial stress compared to participants from other regions (*p* = .021) (Table 2). On the other hand, participants from Northwest China were also significantly more likely to feel horrified due to the COVID-19 pandemic (i.e., following the re-opening of the Wuhan city) than participants from other regions of China (*p* = .030).

Participants aged 40–49 years and ⩾ 50 years reported a significantly decreased stress from work compared to other age groups (*p* = .007) (Table 3). On the other hand, participants aged ⩾ 50 years reported a significantly increased financial stress and stress from home compared to other age groups (*p* = .028 and 0.015, respectively). In addition, participants aged ⩾ 50 years were more likely to feel hopeless due to the COVID-19 pandemic (i.e., following the re-opening of the Wuhan city) than other age groups (*p* = .025).

Participants with a higher educational qualification level were significantly more likely to experience a decreased stress from work compared to participants with a lower educational qualification level (1.6 vs. 1.8) (*p* = .034) (Table 3). Although participants with a higher educational qualification level were significantly more likely to feel horrified due to the COVID-19 pandemic than participants with a lower educational qualification level (3.5 vs. 3.2) (*p* = .010), participants with a lower educational qualification level were significantly more likely to feel helpless due to the COVID-19 pandemic (i.e., following the re-opening of the Wuhan city) (3.6 vs. 3.3) (*p* = .001).

In terms of marital status, participants who were married were significantly more likely to experience less stress from work than participants who were single (1.5 vs. 1.8) (*p* < .001) (Table 4). However, participants who were married were significantly more likely to experience financial stress than participants who were single (3.3 vs. 3.1) (*p* = .005). In addition, participants who were married were significantly more likely to feel horrified, apprehensive and helpless (3.6, 3.5 and 3.4, respectively) due to the COVID-19 pandemic (i.e., following the re-opening of the Wuhan city) than participants who were single (3.4, 3.3 and 3.2, respectively) (*p* = .008, 0.015 and 0.005, respectively).

Participants who had a part-time job were significantly more likely to experience increased financial stress and stress from home and feel apprehensive due to the COVID-19 pandemic (i.e., following the re-opening of the Wuhan city) than participants who had a full-time job and were students (all *p* < .05) (Table 4). On the other hand, participants who had a full-time job were significantly more likely to feel horrified due to the COVID-19 pandemic (i.e., following the re-opening of the Wuhan city) than participants who had a part-time job and were students (*p* = .014).

## Discussion

To our knowledge, our study was the first study to investigate the psychological impact and lifestyle changes of COVID-19 among the general population in mainland China immediately after the travel restrictions in the Wuhan city were lifted on 8 April 2020. The re-opening of the Wuhan city by the Chinese government reflected a significant milestone in the fight against COVID-19 in China. This is because due to the continuous spread of COVID-19, several strict isolation measures and delays had been previously implemented in workplaces, schools and universities in China (Cao et al., 2020). In addition, some families might encounter some financial difficulties because of losing their income sources amid the lockdown in the Wuhan city (Cao et al., 2020). With the re-opening of the Wuhan city, healthy residents are now allowed to move in and out of the Wuhan city, especially for work purposes. University students are also allowed to travel back for continuing their education. However, there are no detailed studies that have investigated the mental health status of the general population after the Wuhan city was re-opened. Therefore, our study provided a unique opportunity to compare the psychological responses and lifestyle changes in the Chinese population in the later stages of the pandemic period with the findings reported in our previous study which was conducted during the early stages of the pandemic period (i.e., between January and February 2020) (Zhang & Ma, 2020).

In our study, the overall mean IES score in participants was 21.5, which was categorised as mild stressful impact amid the COVID-19 pandemic (i.e., following the re-opening of the Wuhan city). The overall mean IES score reported in our current study was higher than the overall mean IES score reported in our previous study conducted during the early stage of COVID-19 pandemic (21.5 vs. 13.6, respectively) (Zhang & Ma, 2020). A study by Wang et al. reported that the broadcasting of health information regarding the COVID-19 pandemic to increase public awareness via radio was associated with increased levels of depression and anxiety in a group of Chinese participants (Wang, Pan, Wan, Tan, Xu, McIntyre, et al., 2020). Therefore, a higher IES score reported in our study was possibly because our participants had been repeatedly exposed to stressful media messages regarding the COVID-19 pandemic for the extended period of several months until now ( Wang, Pan, Wan, Tan, Xu, McIntyre, et al., 2020). According to the COVID-19 situation reports by the WHO, as of 7 May 2020, there have been more than 3.5 million confirmed cases of COVID-19 worldwide, including more than 250,000 deaths (https://www.who.int/emergencies/diseases/novel-coronavirus-2019/situation-reports/). In addition, participants might also feel that they could do little to change the current external situation. Taken together, these factors could have possibly played an important role in increasing the stressful impact experienced by our participants.

In addition, our study reported that 25.5% of the participants had an IES score ⩾ 26 amid the COVID-19 pandemic (i.e., following the re-opening of the Wuhan city), which was higher than that of our previous study (7.6%) (Zhang & Ma, 2020). It is suggested that as time passes, the stress levels among our participants might have been increased significantly, especially after the Chinese Spring Festival. This is because there was increased stress and concern over job security following job cuts due to the COVID-19 pandemic. Although it is still uncertain when the COVID-19 pandemic would come to an end, the sudden incidence of such dramatic crisis caused by the COVID-19 pandemic would cause some behavioural changes among the general population in mainland China. Therefore, further research is warranted to investigate how these behavioural changes happen in the face of major disaster events including the COVID-19 pandemic.

Although none of our participants or their family members/friends were positive for the COVID-19 disease, it is possible that the COVID-19 pandemic has led to significant life stress in their daily routine. Our study reported that female participants had a significantly higher financial stress and stress from home than male participants (all *p* < .05). In addition, our study also found that female participants were also significantly more likely to feel horrified due to the COVID-19 pandemic (i.e., following the re-opening of the Wuhan city) than male participants (*p* < .05). This is because females who were usually the family caregivers would experience increased caregiving responsibilities amid the COVID-19 pandemic. Therefore, they were more likely to experience more emotional stressors than males (Sharma et al., 2016).

Our study also reported that participants who were married had significantly increased financial stress and stress from home compared to participants who were single (all *p* < .05). In addition, participants who were married were significantly more likely to feel horrified, apprehensive and helpless due to the COVID-19 pandemic than participants who were single (all *p* < .05). One possible reason was that marriage might be an independent risk factor for some of these negative impacts. There are several dimensions of marriage including childbearing, courtship, reproductive health and relationships with the family members. Moreover, it is also important to note that domestic violence could have taken place in a marriage during the COVID-19 pandemic. Therefore, these negative impacts on mental health might contribute to the higher IES score in our study. Future studies should investigate if there is increased domestic violence among the vulnerable populations following the mandatory lockdowns due to the COVID-19 pandemic (Usher et al., 2020).

Our study had several unique strengths. Our study was the first study to investigate the immediate impact of the COVID-19 pandemic following the re-opening of the Wuhan city in April 2020. This is particularly imperative because our study provided some important data regarding the psychological responses and lifestyle changes, especially during the later stages of the COVID-19 pandemic. Although convenience sampling was used for participant recruitment, our study included a reasonable sample size from each region of China to ensure that our findings could be generalised to the whole Chinese population in mainland China. Participants were also asked to indicate if they contracted the SARS-CoV-2 or they had family members and/or friends who has tested positive for COVID-19. One limitation of our study was that since these psychological impacts were self-reported, it is possible that these psychological impacts might be not exclusively aligned with the objective evaluation conducted by mental health professionals. Therefore, our findings should be interpreted cautiously.

## Conclusion

In conclusion, our study reported that the COVID-19 pandemic (i.e., following the re-opening of the Wuhan city) was associated with increased stressful impact in our participants than our previous study. The increasing stressful impact following the COVID-19 pandemic (i.e., following the re-opening of the Wuhan city) should not be taken lightly. It is imperative that the Chinese government should consider taking some immediate public health interventions to ease such negative health impacts among the general population in mainland China.
